# Comparative Analysis of the Classification of Food Products in the Mexican Market According to Seven Different Nutrient Profiling Systems

**DOI:** 10.3390/nu10060737

**Published:** 2018-06-07

**Authors:** Alejandra Contreras-Manzano, Alejandra Jáuregui, Anabel Velasco-Bernal, Jorge Vargas-Meza, Juan A Rivera, Lizbeth Tolentino-Mayo, Simón Barquera

**Affiliations:** 1 Center for Research on Nutrition and Health, National Institute of Public Health, Cuernavaca 62100, Mexico;. alejandra.contreras@insp.mx (A.C.-M.); anabel.velasco@insp.mx (A.V.-B.); jorge.vargas@insp.mx (J.V.-M.); mltolentino@insp.mx (L.T.-M.); sbarquera@insp.mx (S.B.); 2 National Institute of Public Health, Cuernavaca 62100, Mexico; jrivera@insp.mx

**Keywords:** nutrient profiling system, NPSC, Health Star Rating, PAHO model, multiple traffic light, Mexican Nutrition Seal, Chilean Warning Octagons, critical nutrients, ultra-processed products

## Abstract

Nutrient profiling systems (NPS) are used around the world. In some countries, the food industry participates in the design of these systems. We aimed to compare the ability of various NPS to identify processed and ultra-processed Mexican products containing excessive amounts of critical nutrients. A sample of 2544 foods and beverages available in the Mexican market were classified as compliant and non-compliant according to seven NPS: the Pan American Health Organization (PAHO) model, which served as our reference, the Nutrient Profiling Scoring Criterion (NPSC), the Mexican Committee of Nutrition Experts (MCNE), the Health Star Rating (HSR), the Mexican Nutritional Seal (MNS), the Chilean Warning Octagons (CWO) 2016, 2018 and 2019 criteria, and Ecuador’s Multiple Traffic Light (MTL). Overall, the proportion of foods classified as compliant by the HSR, MTL and MCNE models was similar to the PAHO model. In contrast, the NPSC, the MNS and the CWO-2016 classified a higher amount of foods as compliant. Larger differences between NPS classification were observed across food categories. Results support the notion that models developed with the involvement of food manufacturers are more permissive than those based on scientific evidence. Results highlight the importance of thoroughly evaluating the underlying criteria of a model.

## 1. Introduction

Worldwide, obesity and chronic disease rates have increased alarmingly in the past 20 years [1]. The combined prevalence of overweight and obesity in Mexican adults was 72.5% in 2016 [2], leading the second place in obesity globally. The prevalence of diabetes (Type-1 and Type-2) was of 15.9% in 2011 [3], by far the highest among The Organisation for Economic Co-operation and Development (OECD) countries [4]. In response to the obesity epidemic, the World Health Organization (WHO) has focused on helping governments enable public policies aiming to improve food environments by reducing the availability and affordability of unhealthy foods [5,6,7,8,9]. In this context, Mexico has implemented regulations on advertising, including voluntary (i.e., the Mexican Nutritional Seal) and mandatory (i.e., Guideline Daily Allowances) front-of-package labeling, and fiscal policies on sugar sweetened beverages as part of a government led national strategy for the prevention and control of overweight, obesity, and diabetes [10]. However, the nutritional criteria used by these strategies are of concern since some of the existing criteria may not be helpful in identifying processed and ultra-processed products containing excessive amounts of sodium, free sugars, saturated fat, total fat, and trans-fatty acids, those are which are associated with an increased risk for chronic disease when consumed in high levels [11]. 

Since most of the actions taken by governments to address obesity are focused on diet as one of the key determinants of disease, it is required to define and classify food and drink products containing excessive amounts of such nutrients. One way to establish acceptable critical nutrient thresholds is with a nutrient profiling system (NPS) [5]. WHO defines nutrient profiling as “the science of classifying or ranking foods according to their nutritional composition for reasons related to preventing disease and promoting health” [5]. Nutrient profiling is a tool to categorize foods, not diets, and national authorities can use them to formulate and apply policies like marketing restrictions to children, product labeling, or health and nutrition claims [12]. 

Overall, NPS can help promote public health dietary goals ensuring consistency with national objectives for non-communicable disease prevention. To fight obesity, chronic diseases and promote health, regional criteria for NPS have been developed with the help of experts in nutrition in different WHO Regions, including PAHO [11]. Despite these efforts, there is still a widespread use of other NPS around the world, including some developed with the participation of the food industry which has been recognized as problematic due to conflicts of interests [13,14]. 

Validation is defined as “the capacity of a nutrient profiling to compile the dietary habits of a population and, through this information, to be able to recognize foods whose recommendations are easy to understand by consumers in order to choose and to put on their tables healthier (or less harmful) food products” [15].This is important given that not every NPS is designed for one purpose or population and thus is not necessarily transferable to another; in addition, the proliferation of different NPS could cause confusion or misinterpretation [5]. WHO has proposed some methods to validate NPS, including calibration (comparing the classifications of one NPS with those from a validated NPS that has been designed for similar purposes), indicator foods method (using a small number of foods previously identified as either “healthy” or “unhealthy” by reference nutrition professionals or food-based dietary guidelines, and assess whether classifications using the NPS agree with the predetermined classifications of these foods) or construct validity (testing whether healthy foods identified by the NPS make healthy diets as defined by an independent, preferably validated dietary quality index, or whether unhealthy foods make unhealthy diets) through, for example, cohort studies, which give the best study design for validation [12]. Other validation methods include content validity, face validity, feasibility, discriminant validity, convergent validity, criterion validity and predictive validity [16]. Although they are useful, they differ in their relative importance and relative costs. Despite the high availability of validation methods, none of them have been established as the gold standard [17], compromising the quality of the validation methods used so far [18,19,20,21,22]. To date, at least thirty NPS have been developed around the world but few of them have been robustly validated [12,15,16,17,18,19,20,21,22,23]. 

Recently, NPS have been implemented in Latin America. For example, in June 2016 Chile introduced a compulsory front-of-pack warning label for food products exceeding specified limits of sodium, sugar, energy, and saturated fats [24]. The enforcement of the label was considered an initial NPS with consequent reductions of the nutritional criteria at 24 (2018) and 36 (2019) months after the application of the label. Ecuador also implemented a NPS for their Multiple Traffic Light (MTL) front-of-pack label [25].

It is recommended that NPS are objective, transparent and reproducible because of their importance in their application in front of pack labeling, restrictions on selling or marketing, health and nutrition claims, and other known uses [6]. For example, the PAHO model has one of the strictest criteria. To our knowledge, few studies have compared the application of different NPS across settings [26,27,28]. The objective of this paper is to compare the ability of the PAHO model and other NPS to identify processed and ultra-processed products containing excessive amounts of critical nutrients (sodium, total sugars, added sugars, saturated fat, total fat, and trans fat), applying their criteria in a sample of products commercialized in Mexico. A secondary objective was to compare the content of critical nutrients between the products that comply or do not comply with the criteria of each NPS. 

## 2. Materials and Methods

### 2.1. Database of Food Products Available on the Mexican Market

Processed and ultra-processed food products were defined according to the PAHO model as “food products manufactured by industry in which salt, sugar, or other culinary ingredients have been added to unprocessed or minimally processed foods to preserve them or make them more palatable” [11]. Most of the processed foods have 2 or 3 ingredients, while ultra-processed foods have 5, 10, 20 or more items including substances that are extracted from foods but have no common culinary use; substances synthesized from food constituents; and additives used to modify the color, flavor, taste, or texture of the final product [11].

Data on the nutritional content of processed or ultra-processed foods available on the Mexican market was collected between 2015–2016 according to Kanter et al., 2017 [29] methods for measuring packaged food and beverage products in supermarkets. Fieldworkers were nutritionists trained during a one-month workshop [30] in which specific didactic training on the food composition, food labeling, and food promotion of packaged foods were revised. In addition, fieldworkers received one day of photography training by a professional photographer and half a day of field-work training in a supermarket. Finally, they were trained and standardized to capture the collected information in the database.

Data collection was conducted in the four cities with the highest population density and economic relevance in the country: Ciudad de Mexico, Guadalajara, Monterrey, and Queretaro. Additionally, we purposely included other smaller cities to capture the diversity of foods available in urban cities compared to different regions of Mexico, especially in the north of the country where a wider variety of products are usually available due to their proximity to the United Sates. These cities include: Cuernavaca, Baja California, Saltillo, and Ciudad Juárez. Altogether, these cities represented approximately 28% of the Mexican population in 2015 [31]. 

A sample of 127 retail and convenience stores located in the census tracts with the highest population densities within each city were selected. The total amount of stores measured within each city varied between 3 in Cuernavaca and 65 in Mexico City, depending on the population and size of the city. We included hypermarkets, supermarkets, convenience stores, and membership food stores from the top grocery retailers in Mexico, which altogether represent more than 70% of the market share in the country [32]. 

All food products in selected food categories available at the time were included. The data collected by the fieldworkers includes product information (e.g., company, brand), container size, price, nutrient facts panel information, ingredients list, nutritional claims, front-of-pack labeling, and photos of all sides of the packages. Nutritional information was recorded for products in the “as sold” form, and if needed (e.g., beverage powders), values for the “as consumed” form were retrieved from the photos of the products. The information contained in each photograph was captured by fieldworkers using a capture mask in the software Research Electronic Data Capture (RedCap, Vanderbilt University, Nashville, TN, US) developed by a multi-country institutional research team and a field supervisor revised the completeness and accuracy of the captured data.

For this analysis, foods and beverages were classified according to their nutritional composition following NOVA food classification [33]. For the present analysis we selected food groups which commonly include processed or ultra-processed products and have been previously used for the discussion of food nutrition policies [34]: dairy products (except cheese), non-dairy beverages, salty snacks, breakfast cereals, and ready-made foods were included, comprising a total of 5996 food products. The database was transferred to STATA (version 13, StataCorp, College Station, TX, USA) format to be reviewed, completed, and cleaned. First, we verified that the nutrient content of products in the database was correct by identifying outliers and corrected the information when appropriate using the photographs of each product. Then, we verified missing information for specific nutrients. When information for specific nutrients (i.e., trans fat or added sugar) was not reported in the nutrients facts panel, its content was calculated when possible. For example, if added sugar was not listed in the nutrients fact panel, the content of this nutrient was determined to be 0 if the content of total carbohydrates was 0 or if no added sugars were declared in the ingredients list. For other cases (*n* = 1886) the content of added sugars was estimated using the method proposed by PAHO [11], which recommends considering added sugars as equal to total sugars if the product is part of a group with no or a minimal amount of naturally occurring sugars, 50% of declared total sugars in yogurt, milk or processed fruit products with sugar in the list of ingredients, and added sugars equal to 75% of declared total sugars if the product has sugar, milk or fruit in the list of ingredients (e.g., cereal bars with fruit). Missing values on poly- (*n* = 1038), mono- (*n* = 1042), trans- (*n* = 1009) or saturated-fats (*n* = 33) were determined to be 0 if the content of total or saturated fat was 0. 

### 2.2. Nutrient Profiling Systems

We compared seven different NPS that are used for front-of-pack labeling based on the fact that they have been relevant for the debate of nutritional criteria in Mexico. It is important to note that the PAHO model is considered the reference NPS because it is based on robust scientific evidence, has been previously validated [27], and is the result of rigorous work by an Expert Consultation Group composed of recognized authorities in the field of nutrition in Latin America. 

The seven NPS included in the comparison are: the Pan American Health Organization (PAHO) model [11], the Nutrient Profiling Scoring Criterion (NPSC) model [35], as well as the NPS used to classify food products by the Australian Health Star Rating (HSR) [36], the Mexican Nutritional Seal (MNS) [37], the Chilean Warning Octagons (CWO) (including the 2016 or most permissive, as well as the 2018 and 2019 criteria) [25,38,39], and the multiple traffic light model in Ecuador (MTL) [39]. We also included a profile proposed in 2010 by a Mexican Committee of Nutrition Experts (MCNE,) independent from the food sector [40]. Details of the criteria for each NPS were reviewed and collected from the official websites and documents (Table 1). 

### 2.3. Calculation of Nutrient Profiling Systems

**Figure 1:**
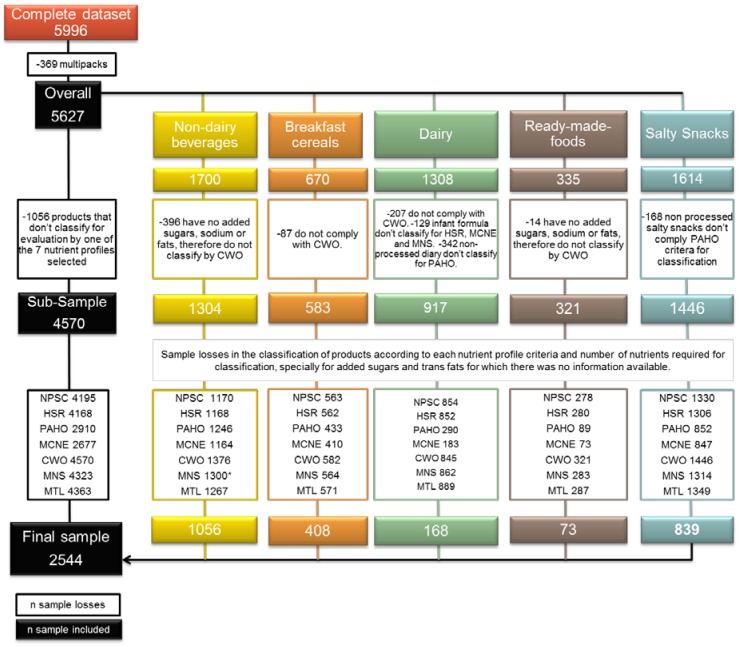
Dropouts of the sample. PAHO = Pan American Health Organization, HSR = Health Star Rating, MTL = Ecuador’s Multiple traffic light, MCNE=Mexican Committee of Nutrition Experts, MNS = Mexican Nutrition Seal, NPSC = Nutrient Profiling Scoring Criterion, CWO = Chilean Warning Octagons (Source: Elaborated by the authors).

From the 5996 products included in the dataset, a total of 3452 were excluded from the analysis because they could not be ranked by all NPS (e.g., raw products are not considered by PAHO) or because of missing information on critical nutrients. However, although 100% juices or nectars are not considered as compliant by the PAHO model (*n* = 255) and sugar sweetened beverages (*n* = 1195) and salty snacks (*n* = 839) are excluded from the Mexican Nutrition Seal algorithm, we kept these food products in the final sample for comparison purposes. The detailed breakdown of the products excluded is described in Figure 1.

According to each NPS, the nutrient profiles of processed foods were calculated independently by two researchers using algorithms generated in STATA. The detailed process for their application to the database is provided in Supplementary Material. A comparison between the results obtained by each researcher was conducted and any disagreements were resolved by discussion with the research team. Then, 30 products were randomly selected and scores calculated by the algorithms were compared to manual calculations. This process was repeated until results matched 100% for all the nutrient profiles.

Food products were classified as compliant or not compliant, based on the nutritional criteria established by each NPS. For the NPSC, a product was considered as compliant when it met the predefined cutoff scores, which vary depending on the NPSC food category; for the HSR, when the product obtained five stars; for the MTL, when all nutrients considered by the algorithm (total fat, sugar and salt) were classified as green; for the CWO, when the product was not eligible for any warning label according to the 2016, 2018 and 2019 criteria (i.e., it did not exceed any of the limits of the nutrients evaluated by the NPS: energy, sodium, sugar, and saturated fat); and for the PAHO model, MCNE, and the Mexican Nutritional Seal, when it complied with all the stipulated criteria.

### 2.4. Nutrient Profiling System Outcome Analysis

The ability of NPS to identify processed and ultra-processed products containing excessive amounts of critical nutrients was determined by the number and proportion (percentage and 95% CIs) of food products classified as compliant, overall and by food category, for each selected NPS. Differences in the proportion of compliant foods between the PAHO model and each NPS were explored using tests of proportions. We also calculated the mean (SD) content of critical nutrients (energy, proteins, total fat, saturated fat total sugars, added sugars, sodium and fiber) of products classified as compliant and non-compliant, by food category for each selected NPS. 

We included the 2016–2019 criteria for the CWO, as well as a ≥3.5 stars criteria for the HSR, for comparative purposes. Linear regression models were estimated to test differences in the mean content between compliant and non-compliant products, by food category for each selected NPS. For all the analyses, significance was established when *p* < 0.05. Analyses were performed using STATA.

## 3. Results

Food categories and classification of the 2544 products included in the study (1056 non-dairy beverages, 408 breakfast cereals, 168 dairy products, 73 ready-made foods, and 839 salty snacks) are presented in Table 2. 

The ability of the models to identify processed and ultra-processed products containing excessive amounts of critical nutrients varied considerably between NPS (Table 3). Overall, the PAHO model classified only 2.3% of products as compliant. No differences in the proportion of foods classified as compliant were observed between the PAHO model and the 5-star criteria of the HSR (2.9%). The rest of the NPS classified a higher proportion of foods as compliant (*p* < 0.05), ranging from less than 5.4% and 6.5% of all foods for Ecuador’s MTL and the MCNE, to 24.1%, 18.9% and 17.2% according to the NPSC, the MNS, and the 2016 criteria of the CWO, respectively. Similar differences were also observed in the proportion of compliant foods by food category. No differences were observed in the proportion of compliant foods between the PAHO model and 5-star criteria HSR for most food categories, except for ready-to-eat cereals (PAHO: 1.7% vs. HSR: 4.2%, *p* < 0.05) and salty snacks (PAHO: 1.0% vs. HSR: 2.4%, *p* < 0.05). Between the PAHO model and Ecuador’s MTL there were also no notable differences, except for beverages (PAHO: 2.3% vs. MTL: 10.7%, *p* < 0.05). In contrast, NPSC consistently classified a higher proportion of foods as compliant compared to the PAHO model for all food categories, especially for dairy products and ready-made foods where almost 50% of foods were classified as compliant (*p* < 0.05). 

Regarding the mean content of critical nutrients among compliant and non-compliant food products (Table 4), the content of most nutrients-to-limit considered in the algorithm of a particular NPS was lower among compliant compared to non-compliant products for all NPS, whereas the content of nutrients-to-encourage (i.e., fiber or protein) was higher (*p* < 0.05). Regarding the MNS, although total sugar was considered as part of the algorithm, no differences were observed in the mean content of this nutrient between compliant (12.7 g) and non-compliant products (12.5 g). 

Differences in the mean content of nutrients not considered in a particular NPS algorithm were also observed for all NPS. For example, although only two NPS considered added sugars in their algorithm (PAHO model and MCNE), the mean content of this nutrient was lower among foods classified as compliant by the rest of the NPS, except the MNS l and the ≥3.5-star HSR. A similar situation was observed for total fat, where most NPS, except the 5 and ≥3.5-star HSR, were able to report lower content of mean total fat among complying products, even when this nutrient was only part of the profiling algorithm of two NPS (PAHO model and Ecuador’s MTL). In contrast, lower mean contents of nutrients-to-encourage were observed among compliant foods for Ecuador’s MTL (fiber: −3.2 g; protein: −5.5 g, *p* < 0.05), the 2016, 2017 and 2018 criteria for the CWO (fiber: from −2.0 g to −3.2 g; protein: from −3.8 to −4.9g, *p* < 0.05), and for MNS (protein: −1.4 g, *p* < 0.05).

## 4. Discussion

Our study showed that the ability of NPS to identify Mexican processed and ultra-processed products containing excessive amounts of critical nutrients varies greatly (i.e., from less than 3% for some food categories to more than 50% for others), depending on the selected model. Overall, the HSR (considering the 5 star criteria), Ecuador’s MTL and the MCNE were able to classify a similar proportion of foods as compliant as the PAHO model, which is considered the reference NPS in our study. In contrast, the NPSC, the MNS and the 2016 criteria of the CWO classified a higher amount of foods as compliant. These differences were even larger when comparing the classification of compliant and non-compliant foods by food category. 

Our results suggest that NPS developed with the involvement of food manufacturers may have less strict criteria than those involving independent academic experts. A NPS with less strict criteria would allow a higher number of foods with a lower nutritional quality to comply with the standards of a “healthy food,” ultimately misinforming consumers and favoring the intake of nutrients-to-limit in the diet of the population [49]. On the other hand, an NPS with very strict criteria would not represent an attractive strategy for the food industry and would limit its effectiveness in the promotion of food reformulation [50]. Evidence suggests that in order for a NPS to set a realistic yet challenging reformulation target, at least 30% of products on the market should meet the NPS criteria at an early stage [51]. From the seven NPS selected, the PAHO model and MCNE were developed by a group of experts with no competing interests. In contrast, the other five NPS have been implemented in different countries, and therefore many of the original set of criteria have been discussed and negotiated with the food industry [52]. Although the criteria established by the PAHO model may be too strict to be implemented in Mexico, where only 2.3% of products would be classified as compliant, it is desirable to eventually have regulations that aim to meet such criteria.

The Chilean front-of-pack labeling experience is a very good example of how labeling policy can be gradually implemented. Although the nutrition criteria of this system are based on evidence, the original proposed regulatory norm was subject to aggressive lobbying opposing the regulation by the food industry [38,39]. The final regulation considered a plan to implement the thresholds progressively in a period of three years, gradually moving nutritional thresholds closer to the criteria established by PAHO [11]. The proportion of foods classified as compliant in our sample was lower when applying the final criteria compared to the 2016 criteria. In contrast, the criteria for the MNS were also negotiated with the food industry [53], however, the nutritional thresholds established are far from the initial or more permissive criteria for the CWO, and do not intend to be aligned to those recommended by PAHO. 

Our study also highlights the relevance of examining the established criteria of NPS used as part of a public health policy. Our comparison of mean nutrient content showed important differences in the content of specific nutrients (i.e., protein and fiber) between compliant and non-compliant foods. For example, despite the fact that the MNS considers total sugar as part of its algorithm, we did not observe differences in the mean content of this nutrient between compliant (12.7 g) and non-compliant products (12.5 g). To explain this inconsistency, we explored this difference within food groups and found that all salty snacks, usually with low contents of total sugar and high protein content, are classified as non-compliant by this NPS, regardless of their nutritional quality. When salty snacks were excluded from the comparison, the mean total sugar in products classified as non-compliant by this NPS was higher (17.5 g) compared to compliant foods (12.7 g, *p* < 0.05) (Data not shown). This also explains in part the lower nutritional quality regarding protein content in non-compliant foods compared to compliant foods according to this NPS. Ecuador’s MTL model is another example of non-desirable protein and fiber differences between compliant and non-compliant foods. This NPS classified a very low number of ready-made foods as compliant (*n* = 3), most of them being ready-to-eat-salads with a low protein content (1.54 g) compared to non-compliant products (8.7 g, *p* < 0.05). As for the higher fiber content among non-compliant foods, when comparing this difference within food products we observed that non-compliant salty snacks had a higher fiber content (+5.4 g, *p* < 0.05, data not shown) compared to compliant snacks (1.4 g, *p* < 0.05). Similar results were observed for the CWO. It is expected that the Chilean and Ecuadorian models are not able to classify foods according to their protein or fiber content, as these models only consider nutrients-to-limit, such as sodium and sugars. 

Although NPS in Ecuador and Chile have been adapted according to public health priorities (i.e., obesity and chronic diseases) considering reductions in the intake of nutrients-to-limit, it is also desirable that the nutritional quality of a diet integrated by products classified as compliant by a particular NPS is aligned with international recommendations for the intake of nutrients-to-encourage [54]. For this purpose, more stringent validation methods would be required, specifically those focused on the effects of NPS on the nutritional quality of the diet [16]. According to a recent systematic review [18], current validation methods for NPS are of low to moderate quality due mainly to the lack of an adequate methodological design to perform the validation. However, rigorous NPS validation methods have been described, which consists of evaluating whether the NPS reflects an individual’s dietary change over time and if this in turn reflects a change in their nutritional and health status, which requires data in at least two points over time and generally with the use of biomarkers and medical records, which make them expensive and impractical for a public health approach [16].

In line with previous literature, our data indicates that some NPS may be more easily adapted depending on the type of model [55]; for example, those based on thresholds, such as the MCNE, MTL, CWO and MNS, may be more challenging to adapt between countries, whereas scoring systems such as the HSR or the NPSC, may establish different score thresholds to suit different purposes. For example, in the case of the HSR criterion with the thresholds established for the 5 stars, the proportion of foods classified as compliant was similar to that of the PAHO model. 

Therefore, we agree with Townsend et al., 2010 [16] and Cooper et al., 2016 [18] in that more studies should be done to establish both the construct and criterion validity for each model of interest, taking into account the context of the target population, such as knowledge of nutrition, vulnerable populations, diet of the population, and availability of food in order to improve public health [34].

Only two models, the NPSC and the HSR, considered the contribution of nutrients or food components to encourage. This may explain in part the higher percentage of foods classified as compliant by NPSC. However, this model has been consistently shown to be more permissive than other NPS [11,35,36]. As for the MNS, results showed that this NPS was one of the least able (similar to CWO 2018–2019) to identify beverages and ready to eat cereals with excessive contents of critical nutrients, especially total sugar, allowing 68% of the juices and nectars and more than a half of the ready-to eat cereals to be classified as compliant. This fact is concerning because juice itself is a source of free sugars and therefore not considered in line with many dietary guidelines [56]. Furthermore, on average, 26.6 g out of a portion of 100 g of ready-to-eat cereals available in the Mexican market is sugar [57]. In the Mexican context, where diabetes is responsible for the death of 100 thousand people annually [58], such permissive criteria should be reconsidered. Adaptations from other NPS that follow the latest international recommendations could be implemented to improve the actual nutrient profile used for the Mexican Nutrition Seal or other dietary policies in Mexico. 

Our study has some strengths and limitations that should be recognized. First, our analyses did not sample food products based on their market share. However, we included a considerable sample of foods sold by the top grocery retailers in Mexico, which account for more than 70% of the market-share in the country. Due to the fact that some NPS select specific foods as non-eligible for profiling (i.e., raw foods), our sample was composed substantially of processed and ultra-processed foods that could be evaluated by the seven NPS, limiting the representativeness of the products analyzed. Foods included relative to foods excluded from our analyses had significantly higher mean content of energy (+35 kcal), total fat (+3.2 g), saturated fat (+1 g), total carbohydrates (+0.8 g), protein (+2.8 g), and fiber (+0.6 g), but lower sodium (−4.4 g, data not shown). Despite this limitation, we believe that by analyzing the same products by all models our study provides comparable data on the ability of NPS to identify food products containing excessive amounts of critical nutrients, as well as their ability to classify foods according to their nutrient quality. Second, although several NPS exist worldwide, we decided to include only those that have been relevant in the debate for nutritional criteria in Mexico. These models could also be relevant for other regions in Latin America, but do not represent a complete view of all NPS available globally. Few studies have compared the use of NPS in low- and middle-income countries and to our knowledge this study is the first to compare the seven selected models [27,28,42]. Another limitation is the fact that due to the diversity of the selected models, the way in which they classify foods is not directly comparable (e.g., HSR assigns between 1 and 5 stars, whereas the PAHO model classifies foods as containing “excessive” or “not excessive” amounts of critical nutrients). In order to make profiling comparable by classifying foods as compliant or non-compliant, we established specific criteria for those models that did not provide a threshold criterion based on the original NPS algorithms.

Another strength of our study was that the PAHO model used as a reference has been previously validated through calibration methods showing higher validity in the identification of foods containing excessive critical nutrients than the WHO euro and the NPSC. Our study also provides calibration data for those NPS not yet validated, such as in the case of the MCNE.

## 5. Conclusions

Our study showed important differences in the ability of NPS to identify processed and ultra-processed products containing excessive amounts of critical nutrients and supports the notion that models developed with the involvement of food manufacturers are more permissive than those developed by experts independent from the food industry [24,27]. Results highlight the importance of thoroughly evaluating the underlying criteria used for front-of-pack food labels and other applications to decrease the consumption of unhealthy products. Although NPS may be useful for classifying commercial products according to the nutrients considered by the model, they may not be that effective in classifying other nutrients to encourage (i.e., proteins and fiber) if they are not included in the NPS algorithm. 

Finally, our results underscore the urgent need to reconsider the nutrient criteria proposed by the Mexican Nutrition Seal. In a context like Mexico, where obesity and diabetes are the leading factors for mortality and morbidity, stringent evidence-based criteria and regulations aligned with international recommendations and other nutrition related policies are needed to promote reformulation by the food industry, especially on nutrients directly related to these diseases (i.e., total and added sugars).

## Figures and Tables

**Table 1:** Nutrient profiling systems description.

Nutrient Profile Scheme/Country	Aim	Food Categories	Foods Not Considered for Classification by the Nutrient Profile	Cut-Off Use	Nutrient Selection	Rationale/Basis */Validation Method	Official Document (Website)
Pan American Health Organization (PAHO) model [11] Countries: Latin American Countries	Provide a tool to classify food and beverages with excess in free sugars, salt, total sugars, saturated fats, and trans fats to be used in the design and implementation of various regulatory strategies.	All processed and ultra-processed foods No categories or food groups	Unprocessed or minimally processed foods: vegetables, legumes, grains, fruits, nuts, roots and tubers, meat, fish, milk, and eggs. Freshly prepared dishes, culinary ingredients (oils, sugar, honey, salt), breast milk substitutes, food supplements, alcoholic beverages	Threshold	(−): Sodium, free sugars, non-nutritive sweeteners, saturated fats, total fats, trans fats	Scientific-Based on WHO recommendations. Validation of PAHO: Calibration [27]	Pan American Health Organization Nutrient Profile Model (http://iris.paho.org/xmlui/bitstream/handle/123456789/18621/9789275118733_eng.pdf?sequence=9&isAllowed=y.)
Health Star Rating (HSR) [36] Country: Australia and New Zealand	Assist consumers to discriminate between foods in the same food category and to compare foods.	All retail food and beverage	Infant formula, food for infants and young children, formulated supplementary sports foods, foods for medical purposes, alcoholic beverages	Scoring	(−) Saturated fat, sodium, sugars (+) Dietary fiber, protein, calcium, certain vitamins and minerals	Regulatory-Based on Food Standards Australia New Zealand (FSANZ) and developed with the collaboration of food industry. Validation of HSR > 3.5 stars cut off point: Calibration [26]	Guide for industry to the Health Star Rating Calculator (HSRC) (http://healthstarrating.gov.au/internet/healthstarrating/publishing.nsf/content/E380CCCA07E1E42FCA257DA500196044/$File/Guide%20for%20Industry%20to%20the%20Health%20Star%20Rating%20Calculator.pdf)
Mexican Committee of Nutrition Experts [41] Country: Mexico	Encourage consumers to select healthier products among the most consumed food groups Stimulate reformulation	Food category 26 food groups: 17 basic groups 7 non-basic groups	Alcoholic beverages, supplements, food products prescribed under medical supervision, and food for infants (<1 year-old)	Threshold	Basic group: (−) Saturated fats, trans fats, added sugar, sodium (+) Fiber Non-basic group: (−) Saturated fats, trans fats, added sugar, sodium, energy (+) Fiber	Scientific-Based on WHO recommendations and Scientific Committee. Not validated.	Report from the Front-of-pack labeling system (FOP-LS) scientific committee to the Mexican Ministry of Health. 2012. Not published
Multiple Traffic Light (Ecuador) [39] Country: Ecuador	To provide clear and precise information about the content and characteristics of processed foods, without being misleading	All processed food for human consumption No categories or food groups	Coffee, tea, aromatic herbs, vinegar, water, salt, alcoholic beverages. Products whose natural content have fat, salt or sugars (with none of these nutrients added), formula and infant food, flours, food additives Packaged produce (fruits, vegetables, chicken, meat, etc.)	Threshold * The cut-off value is different if it is solid (gr) or liquid (ml.)	(−) Total fats, sugars, and salt	Regulatory-Based on Scientific evidence (PAHO, old criteria) in collaboration with industry. Validation of Ecuador’s MTL: face validity [42,43,44]	REGLAMENTO SANITARIO DE ETIQUETADO DE ALIMENTOS PROCESADOS PARA EL CONSUMO HUMANO. (Acuerdo No. 00004522) (https://www.controlsanitario.gob.ec/wp-content/uploads/downloads/2014/08/REGLAMENTO-SANITARIO-DE-ETIQUETADO-DE-ALIMENTOS-PROCESADOS-PARA-EL-CONSUMO-HUMANO-junio-2014.pdf)
Chilean Warning Octagons (CWO) 2016, 2018 and 2019 criteria [24,38] Country: Chile	Provide clear and comprehensive information to the consumer on nutrients that, when consumed in excess, can cause health problems.	Only apply to all national/imported packaged foods & beverages with added sodium, sugars, or saturated fat No categories or food groups	Non-packaged foods and foods that do not have added sugars, saturated fats or sodium	Threshold	(−) Energy, sodium, sugars, saturated fat	Implement the thresholds progressively in a period of three years from most permissive (June 2016) to current (June 2018) to future criteria similar to the PAHO model (June 2019). Validation of CWO: face validity [44]	Reglamento Sanitario de los Alimentos/ Decreto13/2015 (http://www.minsal.cl/wp-content/uploads/2015/08/Directrices-Fiscalizaci%C3%B3n-y-Vigilancia-Decreto-N%C2%BA13_final.pdf)
Mexican Nutritional Seal (MNS) [37] Country: Mexico	Inform general population about foods that comply with nutrition criteria that promote healthy eating.	Food groups 12 categories	Sugar Sweetened beverages, snacks, chocolates, and candy	Threshold	(−) Energy, sodium, saturated fats, total sugars	Regulatory- Not based on scientific or international recommendations. Based on EU Pledge [45,46]. Validation of MNS: Calibration [47].	ACUERDO por el que se emiten los Lineamientos a que se refiere el artículo 25 del Reglamento de Control Sanitario de Productos y Servicios que deberán observar los productores de alimentos y bebidas no alcohólicas pre-envasadas para efectos de la información que deberán ostentar en el área frontal de exhibición, así como los criterios y las características para la obtención y uso del distintivo nutrimental a que se refiere el artículo 25 Bis del Reglamento de Control Sanitario de Productos y Servicios. (http://www.dof.gob.mx/nota_detalle.php?codigo=5340693&fecha=15/04/2014)
Nutrient Profiling Scoring Criterion (NPSC) [35] Country: Australia and New Zealand	Regulation of nutrition and health claims based on its nutrient profile.	All retail food and beverage Three food categories. Includes coconut, spices, herbs, fungi seeds, and algae.	Food that are not for retail and do not require compliance with the Nutrition, Health and Related Claims Standard in the Food Standards Code	Scoring- The final score determines whether a food is eligible to make a health claim	(−) Energy (KJ), saturated fats, total sugars, sodium (+) Fruit/vegetable, nuts, legumes (FVNL), fiber, and protein	Regulatory-Based on UK Food Standards Agency nutrient profiling system and developed with the collaboration of food industry Validation of NPSC: construct [27] and Calibration [48]	Short guide for industry to the Nutrient Profiling Scoring Criterion in Standard 1.2.7—Nutrition, health and related Claims (http://www.foodstandards.gov.au/industry/labelling/Documents/Short-guide-for-industry-to-the-NPSC.pdf)

* Based in terms of threshold value calculation methods and nutritional goals. PAHO = Pan American Health Organization, HSR = Health Star Rating, MTL = Ecuador’s Multiple traffic light, MCNE = Mexican Committee of Nutrition Experts, MNS = Mexican Nutrition Seal, NPSC = Nutrient Profiling Scoring Criterion, CWO = Chilean Warning Octagons. Source: Compiled by the authors.

**Table 2:** Number and proportion of food products overall and by food categories.

Food Category and Classification	*n* Sample	%
**Non-dairy beverages**	**1056**	**41.51**
Powdered beverages	144	13.64
Non-carbonated sugar sweetened beverages with no fruit	254	24.05
Juices and nectars	255	24.15
Beverage concentrates	69	6.53
Carbonated beverages	260	24.62
Energy drinks	11	1.04
Sports beverages	63	5.97
**Salty snacks**	**839**	**32.98**
Fried snacks	502	59.83
Baked snacks	88	10.49
Popcorn	34	4.05
Peanuts	126	15.02
Oilseeds and baked seeds	89	10.61
**Breakfast cereals**	**408**	**16.04**
Ready-to-eat cereal (includes granola and crisped rice)	389	95.34
Oatmeal/amaranth/quinoa-ready-to eat	19	4.66
**Dairy**	**168**	**6.6**
Soy beverages *	67	39.88
Skim milk	1	0.6
Flavored/sweetened/condensed milk	8	4.76
Yogurt drinks	6	3.57
Solid Yogurt	19	11.31
Powdered milk or milk beverages/Cocoa tablets or powder	32	19.05
Milk substitutes /coffee creamer	16	9.52
Reconstituted dairy products or dairy mixes with vegetable fat	5	2.98
Vegetable milk (quinoa, rice, almond, coconut)	9	5.36
Sour Cream	5	2.98
**Ready-made foods**	**73**	**2.87**
Non-processed (salads with dressings and toppings)	6	8.22
Processed (i.e., pizza, sandwiches, hamburgers, burritos)	67	91.78
**Total**	**2544**	**100**

* The food category classification for soy beverages varies depending on the selected nutrient profiling system. In this table all soy beverages are included in the dairy beverages group. Source: Elaborated by the authors.

**Table 3:** Proportion (%) of Mexican foods compliant with nutritional criteria according to seven nutrient profile models, overall and by food category (*n* = 2544).

Nutrient Profile	Overall (*n* = 2544)	Non-Dairy Beverages (*n* = 1056)	Salty Snacks (*n* = 839)	Breakfast Cereals (*n* = 408)	Dairy Products * (*n* = 168)	Ready-Made Foods (*n* = 73)
	% (95% CI)	% (95% CI)	% (95% CI)	% ((95% CI)	% (95% CI)	% (95% CI)
PAHO	2.3 (1.8, 3.0)	2.3 (1.5, 3.4)	1.0 (0.4, 1.9)	1.7 (0.8, 3.6)	9.5 (5.9, 15.0)	5.5 (2.0, 13.8)
5 HSR	2.9 (2.3, 3.6)	2.1 (1.4, 3.1)	**2.4 (1.5, 3.7)**	**4.2 (2.6, 6.6)**	8.9 (5.4, 6.6)	0.0 (0.0, 0.0)
MTL	**5.4 (4.6, 6.4)**	**10.7 (8.9, 12.7)**	0.1 (0.0, 0.8)	0.0 (0.0, 0.0)	12.5 (8.3, 18.4)	4.1 (1.3, 12.1)
MCNE	**6.4 (5.5, 7.4)**	**4.2 (3.11, 5.6)**	0.1 (0.0, 0.9)	**15.7 (12.5, 19.6)**	**29.2 (22.8, 36.5)**	4.1 (1.3, 12.1)
CWO 2019	**10.9 (9.7,12.2)**	**17.4 (15.2, 19.8)**	0.7 (0.3, 1.6)	0.0 (0.0, 0.0)	**36.3 (29.4, 43.9)**	**35.6 (25.4, 47.3)**
CWO 2018	**12.03 (10.8, 13.3)**	**17.4 (15.2, 19.8)**	1.1 (0.1, 2.0)	0.1 (0.0, 2.2)	**42.2 (35.0, 49.9)**	**53.4 (41.9, 64.6)**
CWO 2016	**17.2 (15.8, 18.7)**	**24 (21.6, 26.7)**	1.8 (1.0, 2.9)	**8.8 (6.4, 12.0)**	**46.4 (39.0, 54.0)**	**74.0 (62.7, 82.8)**
≥3.5 HSR	**17.8 (16.4, 19.4)**	3.0 (2.1, 4.2)	**22.9 (20.2, 25.9)**	**36.3 (31.7, 41.1)**	**29.2 (22.8, 41.1)**	**43.8 (32.9, 55.4)**
MNS	**18.9 (17.5, 20.5)**	**16.5 (14.3, 18.8)**	0.0 (0.0, 0.0)	**50.9 (46.1, 55.8)**	**51.2 (43.6, 58.7)**	**19.2 (11.7, 29.9)**
NPSC	**24.1 (22.5, 25.7)**	**20.7 (18.4, 23.3)**	**17.3 (14.9, 20.0)**	**33.1 (28.7, 37.8)**	**47.6 (40.2, 55.2)**	**46.6 (35.4, 58.1)**

PAHO = Pan American Health Organization, HSR = Health Star Rating, MTL = Ecuador’s Multiple traffic light, MCNE = Mexican Committee of Nutrition Experts, MNS = Mexican Nutrition Seal, NPSC = Nutrient Profiling Scoring Criterion, CWO = Chilean Warning Octagons. * Does not include cheese; soy beverages were included in the dairy product category according NPSC, HSR, and MNS classification. Numbers in **bold** indicate significant differences (*p* < 0.05) in comparison with the PAHO model. Compliance was defined as: NPSC; when the product was classified as “healthy” according to NPSC profile, HSR: when the product obtained 5 stars. PAHO, MCNE and MNS: when the products were compliant with all the criteria stipulated by the profile. MTL: when the product classifies the three critical nutrients as green. Chilean Warning Octagons: when the products exceeded any of the limits for critical nutrients. MNS considers SSB and Salty snacks, by definition, not compliant with the criteria. For low energy beverages it was considered if it was compliant with the profile criteria. Source: Elaborated by the authors.

**Table 4:** Mean content of critical nutrients in compliant and non-compliant food products by nutrient profiling system.

	Nutrients-to-Limit						Nutrients-to-Encourage	
NPS	Energy (kcal) (*n* = 2544)	Total Fat (g) (*n* = 2544)	Saturated Fat (g) (*n* = 2544)	Total Sugars (g) (*n* = 2544)	Added Sugars (g) (*n* = 1992)	Sodium (mg) (*n* = 2544)	Fiber (g) (*n* = 2544)	Protein (g) (*n* = 2544)
	Mean (SD)	Mean (SD)	Mean (SD)	Mean (SD)	Mean (SD)	Mean (SD)	Mean (SD)	Mean (SD)
**PAHO model**								
Non-compliant	281.5 (220.4)	**11.4 (15.9)**	**3.1 (5.1)**	12.6 (17.1)	**12.7 (17.5)**	**405.1 (588.9)**	3.2 (5.3)	5.7 (8.6)
Compliant	321.6 (707.2)	**3.2 (4.7)**	**0.6 (1.1)**	10.3 (20.9)	**1.2 (2.9)**	**109.1 (179.2)**	2.2 (3.9)	5.3 (10.2)
**5 HSR**								
Non-compliant	283.7 (243.1)	11.2 (15.7)	**3.1 (5.1)**	**12.7 (17.35)**	**12.6 (17.6)**	**405.1 (590.7)**	**3.0 (5.1)**	**5.5 (8.5)**
Compliant	239.8 (224.2)	12.0 (18.0)	**1.3 (2.3)**	**5.7 (4.9)**	**4.8 (5.3)**	**165.6 (177.6)**	**6.8 (7.7)**	**10.1 (9.3)**
**MTL**								
Non-compliant	**295.6 (241.2)**	**11.9 (16.0)**	**3.2 (5.1)**	**13.2 (17.4)**	**13.3 (17.7)**	**420.0 (593.6)**	3.3 (5.4) *	5.9 (8.7) *
Compliant	**51.6 (124.3)**	**0.2 (0.6)**	**0.1 (0.3)**	**0.8 (1.1)**	**0.8 (1.4)**	**20.2 (21.9)**	**0.1 (0.4)**	**0.4 (1.2)**
**MCNE**								
Non-compliant	**288.4 (245.4)**	**11.8 (16.1)**	**3.2 (5.2)**	**12.7 (17.5)**	**12.9 (17.9)**	**416.2 (597.9)**	**3.0 (5.1)**	5.7 (8.8)
Compliant	**194.3 (176.6)**	**2.8 (4.8)**	**0.1 (0.3)**	**9.2 (11.3)**	**6.9 (8.2)**	**134.1 (175.1)**	**4.7 (6.9)**	4.9 (5.8)
**CWO 2019**								
Non-compliant	**312.6 (239.4)**	**12.5 (16.2)**	**3.4 (5.2)**	**13.8 (17.8)**	**14.0 (18.1)**	**441.5 (604.3)**	3.5 (5.5)*	6.2 (8.9) *
Compliant	**35.1 (56.4)**	**1.0 (2.5)**	**0.2 (0.7)**	**2.4 (2.0)**	**1.9 (1.9)**	**44.8 (83.7)**	**0.3 (0.73)**	**1.3 (2.6)**
**CWO 2018**								
Non-compliant	**314.3 (240.2)**	**12.5 (16.3)**	**3.4 (5.3)**	**13.8 (17.9)**	**13.9 (18.1)**	**443.6 (607.3)**	3.5 (5.5) *	6.2 (8.9) *
Compliant	**47.6 (72.4)**	**1.6 (3.5)**	**0.4 (1.0)**	**2.8 (2.8)**	**2.1 (2.3)**	**64.1 (114.9)**	**0.6 (2.2)**	**1.9 (3.9)**
**CWO 2016**								
Non-compliant	**324.7 (240.9)**	**13.1 (16.5)**	**3.5 (5.4)**	**14.2 (18.3)**	**14.3 (18.6)**	**459.1 (619.2)**	3.5 (5.4) *	6.3 (9.1) *
Compliant	**74.7 (105.6)**	**2.0 (4.4)**	**0.5 (1.1)**	**4.4 (4.8)**	**4.0 (4.9)**	**99.4 (165.8)**	**1.5 (4.4)**	**2.5 (4.3)**
**≥3.5 HSR**								
Non-compliant	**263.1 (243.4)**	9.7 (14.5) *	3.0 (5.3)	**13.0 (18.3)**	12.7 (18.2)	**415.0 (623.4)**	**2.1 (3.8)**	**4.4 (8.2)**
Compliant	371.9 (218.1) *	**18.2 (19.2)**	2.8 (3.9)	**10.1 (10.1)**	10.7 (10.6)	**320.9 (340.5)**	**7.9 (8.0)**	**11.7 (8.0)**
**MNS**								
Non-compliant	**305.1 (251.7)**	**13.2 (16.7)**	**3.6 (5.4)**	12.5 (18.6)	12.5 (19.1)	**460.0 (628.3)**	**2.9 (5.1)**	**5.9 (9.2)**
Compliant	**185.1 (162.2)**	**2.9 (5.2)**	**0.5 (1.0)**	12.7 (8.9)	12.3 (9.5)	**134.1 (165.3)**	**4.3 (5.8)**	4.5 (4.9) *
**NPSC**								
Non-compliant	**298.3 (241.9)**	**11.6 (15.7)**	**3.5 (5.4)**	**14.3 (18.8)**	**14.3 (18.9)**	**466.7 (635.9)**	**2.7 (4.9)**	**5.3 (8.8)**
Compliant	**232.1 (238.3)**	**10.1 (16.0)**	**1.5 (3.1)**	**7.0 (8.4)**	**6.3 (8.3)**	**181.8 (284.5)**	**4.4 (6.1)**	**6.8 (7.8)**

PAHO = Pan American Health Organization, HSR = Health Star Rating, MTL = Ecuador’s Multiple traffic light, MCNE = Mexican Committee of Nutrition Experts, MNS = Mexican Nutrition Seal, NPSC = Nutrient Profiling Scoring Criterion, CWO = Chilean Warning Octagons. Cells in grey indicate nutrients considered in the algorithm of the corresponding NPS. Numbers in **bold** indicate statistical differences (*p* < 0.05) in the mean content between compliant and not compliant foods * indicates difference in the mean content between compliant and non-compliant foods, contrary to what was expected. Source: Elaborated by the authors.

## Supplementary Materials

The following are available online at http://www.mdpi.com/2072-6643/10/6/737/s1.
