# Supplementary material for: Comparative Analysis of the Classification of Food Products in the Mexican Market According to Seven Different Nutrient Profiling Systems
Source: Nutrients. 2018 Jun 7;10(6):737. doi: 10.3390/nu10060737 (PMC6024607; doi:10.3390/nu10060737)
Supplement: Supplementary file 1 [file nutrients-10-00737-s001.pdf]

Annex 1. Application of the 7 NPS algorithms to the Mexican processed and ultra-processed products database.

The data base was made up of 5,996 commercial products from the following 5 food groups: Non-dairy beverages (sugary and unsweetened), salty snacks, breakfast cereals, dairy (except cheeses), and ready-made foods. And 369 multipack products were excluded, leaving a sample of 5,627.

We reviewed the missing values for the nutritional variables of energy, macronutrients, calcium, sodium, fiber, total sugars and added sugars, in addition to the size of the container, number of servings per container, and size of the portion of each product and found 4,589 missing values of added sugars and 3,752 in trans fat.

For added sugars, values were added to the database according to the "Methods for calculating free sugars based on the total sugars declared in food and beverage containers" of panel D of the Profile of the PAHO [1]:

1) 1,023 values "0" were incorporated in the variable of added sugars in products with 0 g of total sugars. Additionally, the value "0" was assigned if no sugars were observed within the ingredients of the product.

For non-dairy beverages, 271 values were added (142 powders / concentrates to prepare beverages, 35 drinks without juice, 20 100% juice and nectars, 51 carbonated drinks, 13 energy or sports drinks). In dairy products, 66 values were added (3 soy beverages, 26 in milk or powder to prepare milk, 2 in solid yogurt, 6 in milk / cream substitutes, 3 in reconstituted milk products, 15 in vegetable drinks, 11 in cream). Furthermore, 40 values were added in ready-to-eat cereals, 47 in ready-made foods, and 609 in salty snacks.

2) 1,612 values were added to added sugars, considering that their quantity is equal to the total sugars declared by the manufacturer if the product was part of a group of foods that do not contain natural sugars or that contain a minimum amount. (For non-dairy beverages we considered "minimum amount" of natural sugars when the product had <15% natural juice.)

For non-dairy beverages, 821 values were added (93 powders / concentrates, 237 drinks without juice, 205 juices and nectars, 258 carbonated drinks and 53 energy or sports drinks), 605 breakfast cereals, 15 dairy products (14 powders to prepare milk drinks, 1 combined dairy product), 30 ready-made foods and 117 salty snacks.

3) 174 values were added to added sugars, estimating their value as 50% of the total sugars when the product was yogurt or milk with sugars in the list of ingredients (106 yogurts, 16 soy drinks and 52 skim milk).

A total of 2,809 were completed in added sugars variables.

For trans-fat, 1642 "0" values were added to the products that did not contain total fat.

Nutrients 2018, 10, 737; doi:10.3390/nu10060737 www.mdpi.com/journal/nutrients

The final sample to analyze was the following:

Table 1. Means of nutrients in 100 g/ml of the products included in the final sample.

Variable	n	Mean	Std. Dev.	Min	Max
Energy (kcal)	5551	139.4	113.7	0	1220
Proteins (g)	5573	3.8	7.7	0	360
Total fat (g)	5584	5.7	10.3	0	200
Saturated fat (g)	5399	1.6	2.7	0	35.5
Trans fat (g)	3360	0.04	0.83	0	33
Carbohydrates (g)	5572	19.7	24.3	0	814
Total sugars (g)	5351	10.7	12.9	0	93.92
Added sugars (g)	3738	8.7	11.8	0	93.92
Fiber (g)	5316	1.5	2.8	0	57.9
Sodium (mg)	5591	154.5	305.3	0	9778.5
Calcium (mg)	947	190.9	191.3	0	1800
Container size (g/ml)	5638	551.7	739.7	3.4	11300
Serving size (g/ml)	5634	115.3	114.0	0.48	1500
Serving per container	5557	11.1	24.7	0.35	511.6

Generation of study variables:

1. The energy content variable in kjoules was generated from the conversion of the energy content in kcal multiply by 4.184.

2. The nutrient value variables were generated per 100 g or ml of the product and another for the nutritional values per container using the variables of "Serving size", "Quantity of X nutrient per serving" and "Container size" through of the following three rules (exemplified by the energy variable):

kcal in 100 g/ml = (100*kcal per serving)/Serving size

kcal per container= (kcal in 100 g/ml * Container size)/ 100

Generation of NPS algorithms:

Nutrient Profiling Scoring Criterion (NPSC): was developed in Australia and New Zealand based on the UK Food Standards Agency nutrient profiling system and target food manufacturers. For this study, the products were classified in two categories: beverages and any food other than those included in the group of beverages [2].

Health Star Rating (HSR): This nutrient profile system is based on the NPSC Criteria and developed for the use of the industry in Australia and New Zealand to determine a qualification for any food or drink other than infant formula, food for infants and young children, formulated supplementary sports foods, foods for medical purposes, alcoholic beverages [3].

Its front labeling was designed to help consumers select and compare foods through 10 different star ratings that can be displayed for foods ranging from ½ star (less healthy) to 5 stars (healthier). For the study sample, the document "Guide for Industry to the HSR Calculator v5 June 2016."[3] was used and the products were classified according to HSR categories such as non-dairy beverages, non-dairy solids, liquid dairy products and other dairy products (such as yogurt) and 129 milk formulas were excluded from the sample in order avoid losing observations due to lack of information in the calcium variable. The "missing" values were replaced by "0" assuming null content [4].

For both the NPS and HSR profiles, a base score was assigned by food category, according to the energy content in kilojoules, saturated fats, total sugars and sodium. Subsequently extra points were assigned for their content of fruit and/or vegetables, fiber and proteins per 100 grams of product. For this step, an exhaustive review of the ingredients of each product in the base was carried out and score V was assigned if it contained fruits, vegetables or seeds (FNLV) in proportion of> 25% for concentrates and> 40% for solid foods.

For the generation of this variable (FNLV) it was considered as a concentrate for beverages when the ingredients were declared "concentrated", "pulp", "juice" or "reconstituted". It was not considered concentrated when it was specified according to the brand, for example; "JUMEX guava / strawberry concentrate", "artificial concentrate", or "flavoring", "concentrate based on essential oils".

The allocation of extra points was carried out in the following way:

A) NON-DAIRY BEVERAGES:

• The score was assigned according to the sum of the percentages referred to in the NFLV ingredients.

• 100% was considered in juices when: 1) 100% of the fruit and / or vegetable concentrate was specified in the name of the product or in the ingredients. 2) If the sum of the first 4 ingredients are concentrated fruit and / or vegetable and results in 100% 3) If only 1 ingredient is found in the product or if it is concentrated, rehydrated, and water. 4) The only ingredient was fruit, vegetables or both.

- A score of> 25% of NFLV was considered in beverages when: reconstituted or pulp was specified in the first 2 ingredients.

B) SALTY SNACKS:

- The score was assigned according to the sum of the percentages referred to in the NFLV ingredients.

- It was considered 100% in snacks when: The sum of the percentage of the ingredients was NFLV, the first ingredient was NFLV and there were no other ingredients or these were salt or oil.

- It was considered 85% of NFLV in Japanese peanuts.

- An assigned score was not considered if the snack contained corn chips.

C) READY-MADE FOODS:

- The score was assigned according to the sum of the percentages referred to in the NFLV ingredients.

- Beans or soybeans were not considered as NFLV, but peas were

- Between60 to 80 percent of NFLV was assigned to salads if it contained dressing, croutons, cheeses, beans, or other non-NFLV ingredients.

- No points were considered for: 1) Seeds in liquid form, aloe, mead. 2) Mixtures in beverages that contained a non-NFLV ingredient and that did not specify% of the product, when the concentrate was in the first 2 ingredients. Except the sports drinks. 3) Mixtures in foods containing a non-NFLV ingredient in the first 2 ingredients.

Finally, the final score was estimated with a sum of the base points and the extra points were subtracted as follows: Points of base nutrients - Points of fiber content - point of content of fruits and / or vegetables - fiber content.

Products complied with the NPSC profile when the final score was less than 1 for group 1 and less than 4 for group 2. 5,152 products of the dataset were classified, of which 34.57% met the criteria.

For the HSR profile, the final score was assigned its corresponding star rating, a total of 4,987 products of the data set were classified, of which 61.8% had less than 3 stars and 7.84% obtained 5 stars.

Pan American Health Organization nutrient profile: Was developed in Latin American Countries to provide policy makers a tool to classify food and beverages with excess in free sugars, salt, total sugars, saturated fats, and trans fats to be used in the design and implementation of various regulatory strategies [1].

First, 410 unprocessed or minimally processed foods (342 dairy products and 68 salty snacks) were identified in order to exclude the sample. 100% juices or nectars are not considered at all as processed foods, but were included in the sample for comparison purposes (n = 193).

Subsequently, the comparison of each critical nutrient with the total calorie content, 1%, 10% and 30% of the total calorie intake was made and compliance was determined. In the case of compliance with trans fats, in those products without information (missing) it was determined that their content was 0 for the purpose of classifying the product.

To know the existence of some non-caloric sweetener in the product, a search was made among the ingredients of the products. For this search, the following terms were considered as sweeteners (caloric or non-caloric): "Sweeteners", "Artificial sweeteners", "Aspartame", "Isomaltosa", "Sucralose", "Acesulfame K", "Stevia", "Acesulfame de Potassium", "Neotame", "Sweetener", "Sorbitol", "Steviosides", "Stevia", "Glucono-noctone", "Steviol", "Inositol" and "Maltodextrin".

Compliance was established when the product met all the cutoff scores of the profile. A total of 3,384 products of the data set were classified according to PAHO model of which 7.18% met the criteria.

Mexican Committee of Nutrient Experts (MCNE): The MCNE profile was developed in Mexico by a Scientific Committee and based on WHO recommendations to stimulate consumers to select healthier products among the most consumed food groups and to stimulate industry to reformulation [5]. MCNE proposes criteria through 7 food groups and in these, 27 food sub-groups. For each subcategory, it details a criterion which specifies the limits of energy, saturated fats, trans fats, sodium, added sugars and fiber.

For this profile, the products were re-categorized in the 7 food groups and 129 milk formulas were excluded. Subsequently, the nutritional criteria corresponding to each group were applied. In order to increase the sample size if there was no available information on trans fat, the product was considered to meet the criteria if the total fat content did not exceed the limit of trans fats per 100 g serving.

Also, this NPS did not establish a specific criterion for soy beverages nor a criteria for juices or nectars. The criteria for juice containing beverages with little energy content was estimated according to the criteria for the School Food Program guidelines for Mexico [6]:

- Soy made liquid foods with or without juice: 70 kcal/100ml.
- Juice: 52 kcal/100ml
- Nectars: 52 kcal/100ml.

- Beverages reduced in energy content: 20kcal/100ml

Of the total sample, 3,189 products could be classified by profile, of which 13.1% met the criteria.

Mexican Nutritional Seal (MNS): The nutritional seal has its basis in the "Agreement by which the guidelines referred to in article 25 of the Sanitary Control Regulation of Products and Services to be observed by producers of pre-packaged foods and non-alcoholic beverages for the purposes of the information that should be displayed in the frontal area of the exhibition, as well as the criteria and characteristics for obtaining and using the nutritional label referred to in article 25 Bis of the Sanitary Control Regulation of Products and Services"[7].

The categorization of foods and beverages was made according to the characteristics dictated by the regulation, 129 dairy formulas were excluded, and by definition all the non-dairy beverages and salty snacks of the criteria because they are considered as unhealthy products, but for comparison purposes, were included in the sample and were classified as "non-compliant".

Subsequently variables for energy were generated considering the portion in which they establish the nutritional criteria using a rule of three:

energy per serving = (reference portion * energy content per serving of the product) / serving size

This was done for each group of food with criteria applicable in energy. Subsequently the same procedure was followed for sodium, saturated fats and total sugars in each food group.

Of the total sample, 5,174 products were classified by the MNS profile, of which 25.4% met all the criteria.

Multiple Traffic Light (Ecuador): Was developed in Ecuador and based on the old PAHO model criteria to provide clear, precise, non-misleading information about the content and characteristics of processed foods was aimed at the general population [8]. It does not consider specific categories or food groups, but excludes coffee, tea, aromatic herbs, vinegar, water, salt, alcoholic beverages, products whose natural content have fat, salt or sugars that have no additives of these nutrients, formula and infant food, flours, and food additives. 129 infant foods formulas were excluded from the sample.

After, we applied the cut-off value for total fats, sugars, and salt depending on if it was solid (gr) or liquid (ml). Compliance of the MTL profile was established when all critical nutrients (total fat, sugar and salt) were classified as green. Of the total sample, 5,211 products we classified by the MTL profile, of which 7.1% had a green color in the three critical nutrients of the profile.

Chilean Warning Octagons (CWO): Were developed in Chile to provide clear and comprehensive information to the consumers on nutrients that,when consumed in excess, can cause health problems. Its target is the general population [9].

It only applies to all national/imported packaged foods and beverages with added sodium, sugars, or saturated fat. For that reason, after reviewing the ingredients of most of the products, we excluded 941 products from the sample.

The profile does not consider categories or food groups, so we applied the cut-off values for energy, sodium, sugars, saturated fat. Compliance was established when the product was not eligible for any warning label (i.e. it did not exceed any of the limits of the critical nutrients).

From the total of the sample, 4,439 products were classified, of which 25.4% met all the NPS criteria.

After applying the algorithms of each NPS, we obtained a subsample of 2,544 products that have the classification of the 7 NPS to compare each other. Detailed description of the dropouts of the sample are shown in Figure 1.

References

1. Pan American Health Organization. Nutrient Profile Model. Washington, DC. 2016. ISBN: 978-92-75-11873-3. Available from: http://iris.paho.org/xmlui/bitstream/handle/123456789/18621/9789275118733_eng.pdf.
2. Food Standards Australia New Zealand (FSANZ). Short guide for industry to the Nutrient Profiling Scoring Criterion in Standard 1.2.7-Nutrition, health and related Claims. Australia and New Zealand: Food Standards Australia New Zealand, 2016. Available from: http://www.foodstandards.gov.au/industry/labelling/Documents/Short-guide-for-industry-to-the-NPSC.pdf
3. Food Standards Australia New Zealand (FSANZ). Guide for industry to the Health Star Rating Calculator (HSRC). Australia and New Zealand 2016. Available from: http://healthstarrating.gov.au/internet/healthstarrating/publishing.nsf/content/E380CCCA07E1E42FCA257DA500196044/$File/Guide%20for%20Industry%20to%20the%20Health%20Star%20Rating%20Calculator.pdf
4. NORMA Oficial Mexicana NOM-051-SCFI/SSA1-2010, Especificaciones generales de etiquetado para alimentos y bebidas no alcohólicas preenvasados-Información comercial y sanitaria. (In Spanish). Available from: http://dof.gob.mx/nota_detalle_popup.php?codigo=5137518.
5. Popkin B BS, Carriedo A. Report from the Front-of-pack labeling system (FOP-LS) scientific committee to the Mexican Ministry of Health. 2012.
6. Secretaria de Salud. Mexico. 2010. Acuerdo Nacional para la Salud Alimentaria. Estrategia contra el sobrepeso y la obesidad. (In Spanish). Available from:

http://www.promocion.salud.gob.mx/dgps/descargas1/programas/Acuerdo%20Original%20con%20creditos%2015%20feb%2010.pdf
7. ACUERDO por el que se emiten los Lineamientos a que se refiere el artículo 25 del Reglamento de Control Sanitario de Productos y Servicios que deberán observar los productores de alimentos y bebidas no alcohólicas preenvasadas para efectos de la información que deberán ostentar en el área frontal de exhibición, así como los criterios y las características para la obtención y uso del distintivo nutrimental a que se refiere el artículo 25 Bis del Reglamento de Control Sanitario de Productos y Servicios. (In Spanish). Available from: http://www.dof.gob.mx/nota_detalle.php?codigo=5340693&fecha=15/04/2014
8. REGLAMENTO SANITARIO DE ETIQUETADO DE ALIMENTOS PROCESADOS PARA EL CONSUMO HUMANO. Acuerdo No. 00004522. (In Spanish). Available from: https://www.controlsanitario.gob.ec/wp-content/uploads/downloads/2014/08/REGLAMENTO-SANITARIO-DE-ETIQUETADO-DE-ALIMENTOS-PROCESADOS-PARA-EL-CONSUMO-HUMANO-junio-2014.pdf
9. Reglamento Sanitario de los Alimentos/ Decreto13/2015. (In Spanish). Available from: http://www.minsal.cl/wp-content/uploads/2015/08/Directrices-Fiscalizaci%C3%B3n-y-Vigilancia-Decreto-N%C2%BA13_final.pdf.
